# “Leave me alone”: anatomical structures and variations seen on computed tomography of the temporal bone

**DOI:** 10.1590/0100-3984.2022.0030

**Published:** 2023

**Authors:** Mehmet H Atalar, Nisa Başpınar, Doğukan Ege Atalar

**Affiliations:** 1 Department of Radiology, Sivas Cumhuriyet University Faculty of Medicine, Sivas, Turkey; 2 Department of Orthodontics, Yeni Yüzyıl University Faculty of Dentistry, İstanbul, Turkey

**Keywords:** Anatomic variation, Tomography, X-ray computed, Temporal bone/diagnostic imaging, Variação anatômica, Tomografia computadorizada, Osso temporal/diagnóstico por imagem

## Abstract

The anatomical structure of the temporal bone is quite complex. There are a great number of anatomical variations that are often confused with temporal bone pathologies, especially fractures. It is important that radiologists and surgeons be able to recognize such variations.

## Abstract (pt)

O osso temporal é uma estrutura anatômica óssea bastante complexa. Apresenta grande número de variações anatômicas, que muitas vezes são confundidas com lesões ou doenças, principalmente fraturas. O reconhecimento dessas variações é importante para radiologistas e cirurgiões.

## INTRODUCTION

The temporal bone is the most complex bony structure in the human body, which makes its radiological evaluation equally complex. The small, thin ossicles, vascular structures, small canals, and thin sutures found in the structure of the temporal bone can pose significant diagnostic challenges in the analysis of temporal bone on computed tomography (CT). In this article, we address the anatomical variations that can pose diagnostic challenges in the CT evaluation of the temporal bone in daily practice.

## TECHNIQUES FOR TEMPORAL BONE CT

The temporal bone contains small, thin anatomical structures. Therefore, image resolution is of utmost importance. On CT, ideal collimation is necessary to achieve high resolution. The slice thickness should be between 0.5 mm and 0.625 mm. The field of view should be 16-22 cm. It is recommended that temporal bone CT involve at least two planes. Axial and coronal views are quite sufficient to delineate the anatomy of the temporal bone and are routinely used for that purpose. Imaging reconstruction is performed parallel to the lateral semicircular canal in the axial plane. Coronal reconstruction is performed perpendicular to the axial plane. The superior semicircular canal should be clearly visualized in the uppermost axial slice. In the coronal view, the most anterior image should be immediately anterior to the geniculate ganglion. In addition to the classic axial and coronal planes, images are now routinely acquired in the Stenvers and Pöschl planes, which are, respectively, parallel to and perpendicular to the long axis of the petrous bone. For temporal bone CT, the window width/level should be 2800/600 or 4000/700^(1,2)^.

Intravenous contrast administration can be helpful for special indications such as complications of otomastoiditis, vascular tumors, and vascular anomalies. However, contrast is rarely necessary for the routine evaluation of mastoid air cells or hearing loss^(1,2)^.

## VASCULAR VARIATIONS

### High jugular bulb

**Figure 1:**
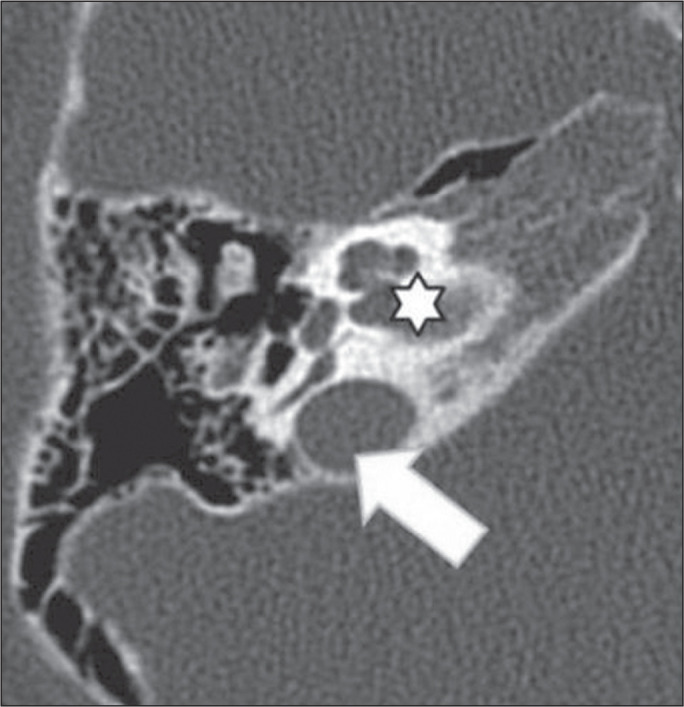
High jugular bulb in a 21-year-old woman. Unenhanced axial CT of the temporal bone, showing a high right jugular bulb (arrow) at the same level as the internal auditory canal (star).

The jugular bulb is the dilated upper end of the jugular vein in the jugular fossa and can be located near the inner ear. Normally, the upper border of the jugular bulb is located under the hypotympanum in the middle ear cavity. The jugular bulb is often asymmetrical. The right jugular bulb is often larger than the left one. Its size and location depends on mastoid bone pneumatization. Absence of a jugular bulb is a normal finding in early childhood. In infants, the jugular bulb is known as the jugular sinus. A high jugular bulb is defined as the extension of the jugular bulb to the posterior semicircular canal, the floor of the internal auditory meatus, or the basal turn of the cochlea (Figure 1). The reported incidence of a high jugular bulb ranges from 4.5% to 8.5%. It is seen equally in both sexes. In cases of a high jugular bulb, different than in those of dehiscence of the jugular bulb, there is a thin bony plate (the sigmoid plate) that separates the jugular bulb from the middle ear cavity^(3-5)^.

A high jugular bulb is usually larger than the contralateral bulb. Although most cases of a high jugular bulb are asymptomatic, symptomatic cases have been reported. It can compress the adjacent structures, causing tinnitus and conductive hearing loss. Symptoms can be aggravated by conditions that increase cardiac output. Patients with a medially located high jugular bulb can present with vertigo, sensorineural hearing loss, or tinnitus. A high jugular bulb can also mimic Ménière’s disease. If a high jugular bulb is suspected preoperatively, radiological evaluation with CT angiography or venography can facilitate the surgical planning and reduce the risk of complications^(3-5)^.

A system for the classification of the jugular bulb position, proposed in 2018 by Manjila et al.^(5)^, describes the relationship between the presence or absence of separations in the internal auditory canal, posterior semicircular canal, and middle ear. This simple and easy-to-apply system is extremely useful for surgical planning in patients with jugular bulb variations.

### Dehiscent jugular bulb

**Figure 2:**
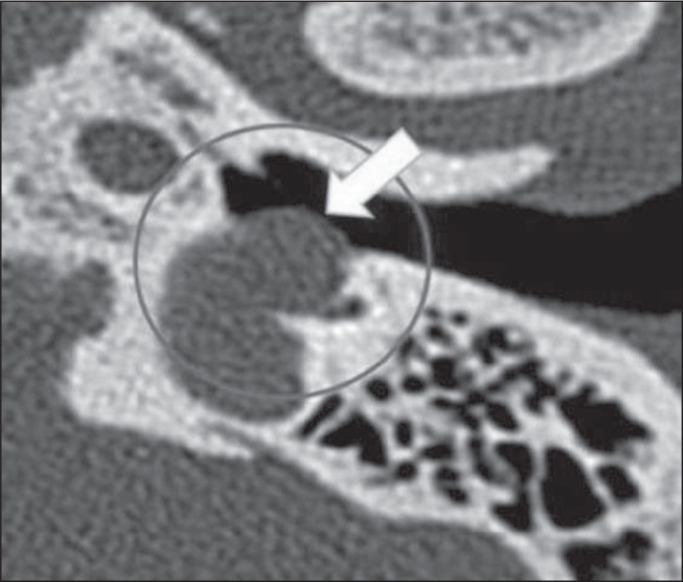
Dehiscent jugular bulb in a 32-year-old woman. Unenhanced axial CT of the temporal bone, showing that the bony plate (arrow) between the left jugular bulb and the middle ear cavity (circle) is not visible.

**Figure 3:**
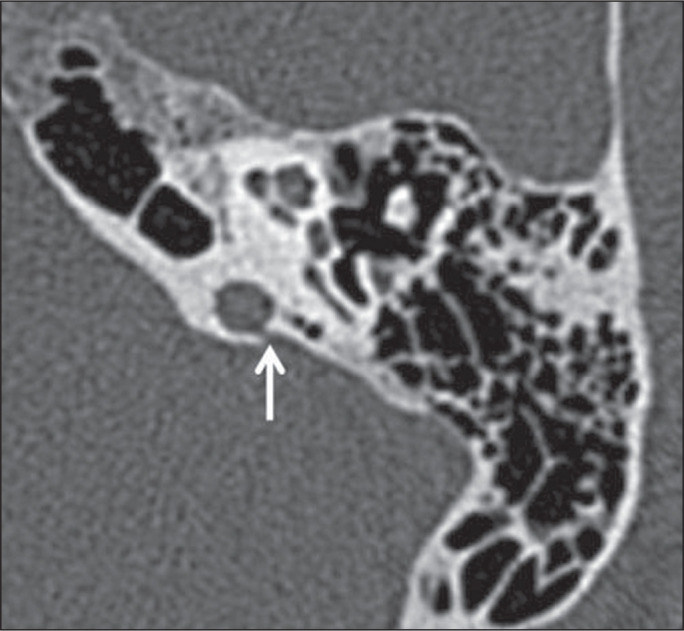
Jugular bulb-vestibular aqueduct dehiscence in a 34-year-old woman. Unenhanced axial CT of the temporal bone, showing no bone plate between the jugular bulb and the vestibular aqueduct (arrow).

**Figure 4:**
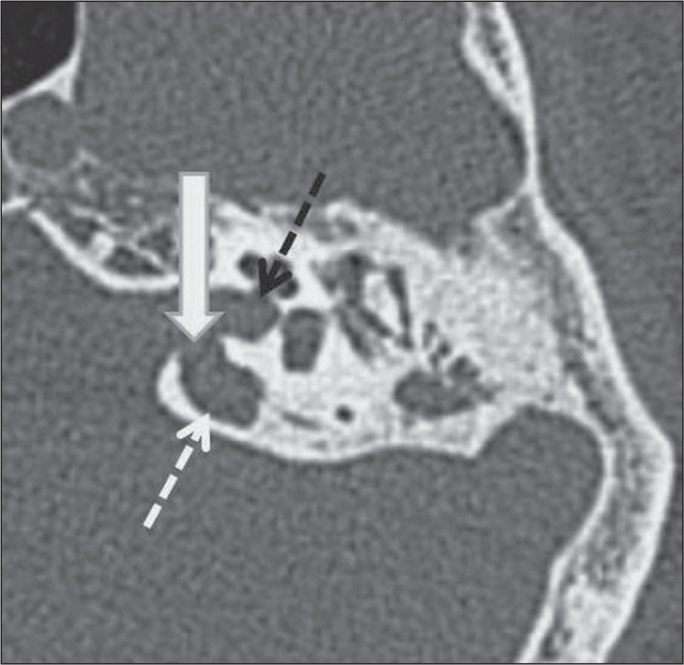
Dehiscence of the jugular bulb and internal acoustic canal in a 35-year-old man. Unenhanced axial CT of the temporal bone, showing no bone plate (white thick arrow) between the jugular bulb (white dashed arrow) and the internal acoustic canal (black dashed arrow). In addition, the jugular bulb (white dashed arrow) is high.

A dehiscent jugular bulb is the normal venous variant of the upper, lateral extension of the jugular bulb from the sigmoid (jugular) sinus to the middle ear cavity. In cases of a dehiscent jugular bulb, there is no bony separation between the jugular bulb and the tympanic cavity (Figure 2). It has an estimated incidence of 5% in the symptomatic population. It is one of the causes of pulsatile tinnitus and is a common cause of retrotympanic vascular mass. Patients with a dehiscent jugular bulb can present with conductive hearing loss. This can occur when the jugular bulb comes into contact with the tympanic membrane or ossicles, as well as when it obstructs the oval window. It is best visualized in coronal CT slices. On unenhanced CT scans, it can be mistaken for glomus jugulare or glomus jugulotympanicum. The dehiscence can also affect the relationship between the jugular bulb and the tympanic cavity, vestibular aqueduct (Figure 3), or internal acoustic canal (Figure 4). A careful analysis of bone margins on CT will exclude more aggressive conditions such as tumors and infection. The use of angiography or venography, performed with CT or magnetic resonance imaging (MRI) plays an important role in the diagnosis of a dehiscent jugular bulb^(4-6)^.

### Jugular bulb diverticulum

**Figure 5:**
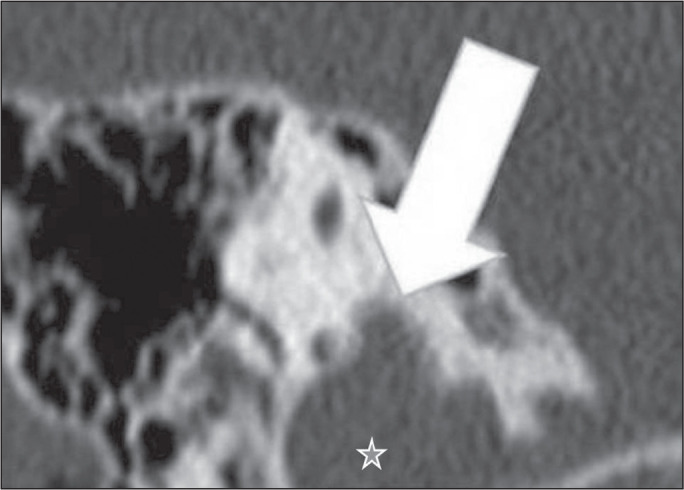
Jugular bulb diverticulum in 38-year-old woman. Unenhanced coronal CT of the temporal bone, showing protrusion of a small diverticulum (arrow) proximal to a high jugular bulb (star).

A jugular bulb diverticulum is a congenital vascular anomaly characterized by a focal, finger-like protrusion extending to the surrounding skull base (Figure 5). It is a markedly cortical, well-defined polypoid extension in the marginal space of the jugular bulb. Its prevalence in imaging studies ranges from 1% to 8%. However, it is defined in cases associated with pulsatile tinnitus, conductive hearing loss, and vertigo. It is an irregular protrusion of the jugular bulb that can extend to the upper surface of the petrous bone, middle ear cavity, endolymphatic canal, or vestibular aqueduct. In rare cases, a jugular bulb diverticulum can erode inner ear structures like the vestibular aqueduct, facial nerve canal, and posterior semicircular canal^(7,8)^.

### Anteriorly located sigmoid sinus

The sigmoid sinus originates at the level of the upper border of the petrous bone, from the junction of the transverse and superior petrosal sinuses. It forms the posterior border of the mastoid bone. Its location within the mastoid cavity can vary, and it can extend to the posterior mastoid. The underlying etiology is unclear. Chronic otitis media in childhood and genetic factors provoking mastoid hypopneumatization can play a role in the etiology. Because the sigmoid sinus can be inadvertently lacerated during mastoidectomy, the presence of an anteriorly located sigmoid sinus should be noted in radiology reports. The distance from the anterior wall of the sigmoid sinus to the posterior wall of the external auditory canal determines the extent of the postauricular surgical approach to the mastoid antrum. The farther the sigmoid sinus is displaced anteriorly, the more difficult it can be to visualize the oval window niche during the planning and execution of a mastoidectomy^(9,10)^.

### Mastoid emissary veins

**Figure 6:**
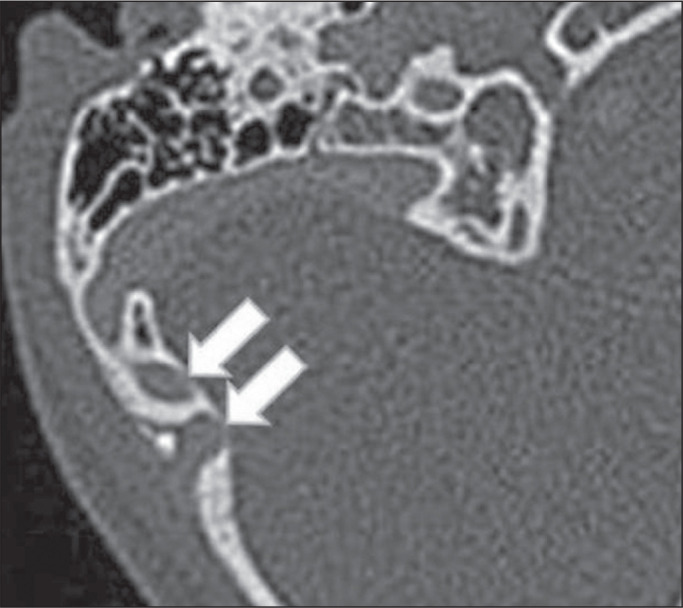
Mastoid emissary veins. Unenhanced axial CT of the temporal bone, showing emissary veins with an intraosseous course (arrows).

Emissary veins are venous structures that pass through the foramina of the skull to establish a connection between the dural venous sinuses and the veins external to the skull. A mastoid emissary vein constitutes a venous variant that should not be mistaken for the lambdoid suture (Figure 6). It passes through the mastoid canal, connecting the transverse or sigmoid sinus to the posterior auricular or occipital vein. It could complicate a retromastoid surgical approach or the insertion of the outer component of a cochlear implant. To avoid potential complications, it is important to have sufficient knowledge of the anatomical relationships and variations of the emissary veins before attempting surgical or endovascular interventional procedures in the posterior cervical region or posterior cranial fossa^(11,12)^.

### Persistent petrosquamosal sinus

The petrosquamosal sinus courses along the petrosquamosal fissure of the temporal bone or within the temporal canal of Vergi. A persistent petrosquamosal sinus is an emissary vein establishing venous communication between the intracranial and extracranial compartments. It unites the external jugular venous system and dural sinuses, draining into the retromandibular vein via the postglenoid foramen or into the pterygoid venous plexus via the foramen ovale. It typically regresses in fetal and early postnatal life. The incidence of persistent petrosquamosal sinus is high in inner ear anomalies, mainly complete semicircular canal aplasia. Its association with **C**oloboma, **H**eart defect, **A**tresia choanae, **R**etarded growth and development, **G**enital hypoplasia, **E**ar anomalies (CHARGE) syndrome has been described. It could pose a risk during cochlear implant surgery or serve a means of spread for septic thrombosis. A persistent petrosquamosal sinus should be mentioned in CT reports. Venous CT angiography or MR venography could be needed for its diagnosis^(13,14)^.

## NONVASCULAR VARIATIONS

### Deep sinus tympani

**Figure 7:**
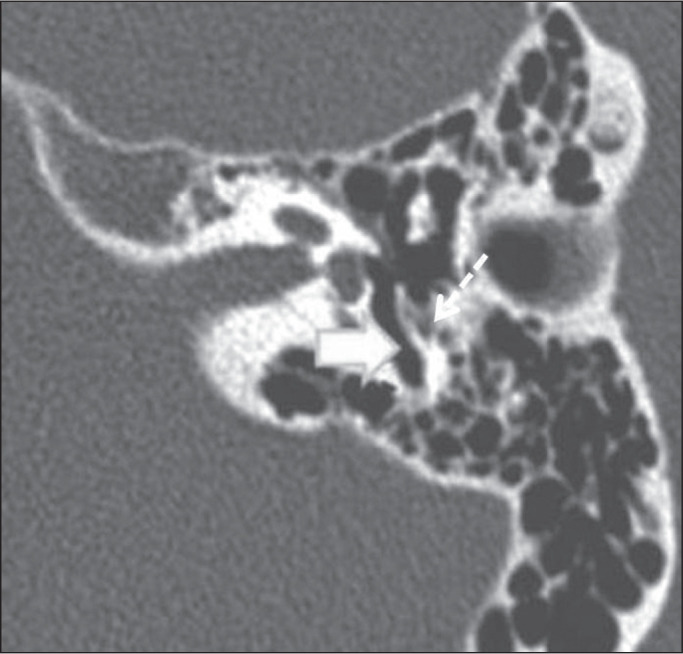
Deep sinus tympani. Unenhanced axial CT of the temporal bone, showing a wide, deep sinus tympani (thick arrow) extending posteromedially to the facial nerve (dashed arrow).

The sinus tympani is a bony recess of the posterior tympanic cavity. It plays a role in the development of chronic middle ear infection and acquired cholesteatoma. The depth of the sinus tympani is categorized, on the basis of the radiological findings^(15)^, as type A (limited), type B (deep), or type C (deep with posterior extension). A deep sinus tympani is defined as having a depth of more than 6 mm on axial high-resolution CT scans (Figure 7) and has an incidence of 5.9%. A deep sinus tympani can be in close proximity to the petrosquamosal sinus canal or facial nerve. For cases in which middle ear surgery (especially cholesteatoma surgery) is considered, the shape and depth of the sinus tympani should definitely be mentioned in the CT report^(15,16)^. A recent study revealed that a deep sinus tympani is more common in children than in adults^(16)^.

### Transverse crest and Bill’s bar

**Figure 8:**
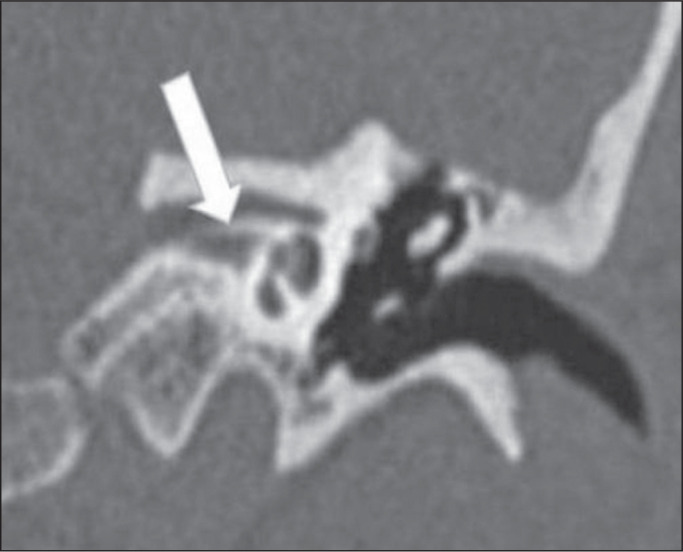
Transverse crest. Unenhanced coronal CT of the temporal bone, showing a horizontal osseous structure (arrow) in the lateral part of the internal acoustic canal, dividing it into upper and lower sections.

The transverse crest is a horizontal protrusion that partitions the internal acoustic meatus into upper and lower segments (Figure 8). It is located in the fundus of the internal acoustic canal and can extend more medially in some patients. It is not typically seen on MRI scans^(17,18)^. Bill’s bar is a vertical protrusion that divides the upper section of the internal acoustic canal into anterior and posterior parts. The facial nerve and intermediate nerve are located in front of Bill’s bar in the anterior-superior quadrant. Behind it, in the posterior-superior quadrant, is the superior part of the vestibular nerve. It is usually impossible to visualize Bill’s bar on a high-resolution CT scan of the temporal bone. However, a clinical study that compared a 7.0-T MRI scanner and a 3.0-T MRI scanner for their abilities to discern the more delicate inner ear anatomy found that Bill’s bar could sometimes be seen at both field strengths^(17-20)^. Together, the transverse crest and Bill’s bar form four quadrants that include the facial nerve in the anterior-superior quadrant, the cochlear nerve in the anterior-inferior quadrant, and the upper and lower segments of the vestibular nerve in the posterior-superior and posterior-inferior quadrants, respectively.

### Cochlear cleft

**Figure 9:**
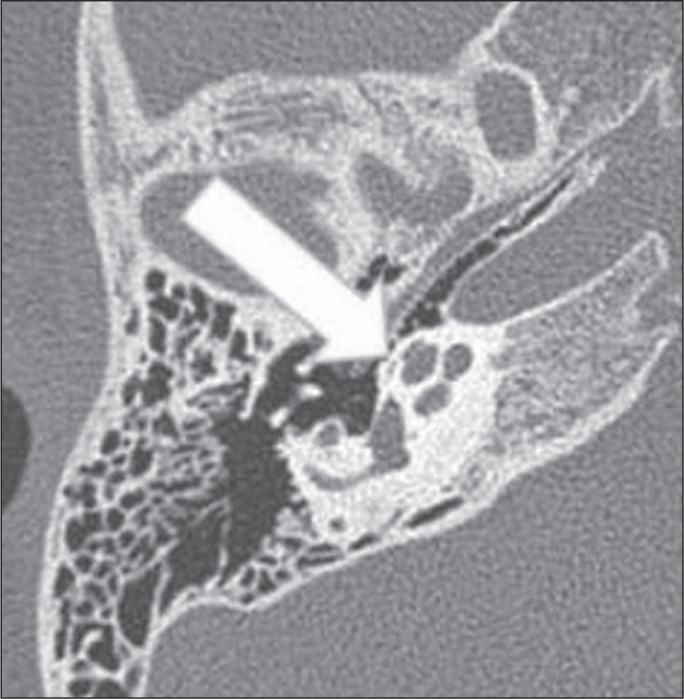
Cochlear cleft in a 15-year-old adolescent male. Unenhanced axial CT of the temporal bone, showing the cochlear cleft in the otic capsule lateral to the middle turn of the cochlea (arrow).

The cochlear cleft is a pericochlear lucency with a narrow curve that extends from the cochlea to the promontorium (Figure 9). It is typically seen in infants and children but can also be found in adults. As seen in front of the oval window on CT, the cochlear cleft is defined as the fatty marrow in areas of incomplete endochondral ossification of the otic capsule. It is C-shaped and can be mistaken for a fracture line or otosclerotic focus. It is related to the fissula ante fenestram. Any curved lucency in the fissula ante fenestram should be considered to suggest “fenestral otosclerosis” in adults, whereas a “cochlear cleft” should be definitely excluded in pediatric patients. On CT, a cochlear cleft is seen as a hypoattenuated, curvilinear focus in the otic capsule around the cochlea^(21,22)^.

### Pseudofractures

#### Occipitomastoid suture

**Figure 10:**
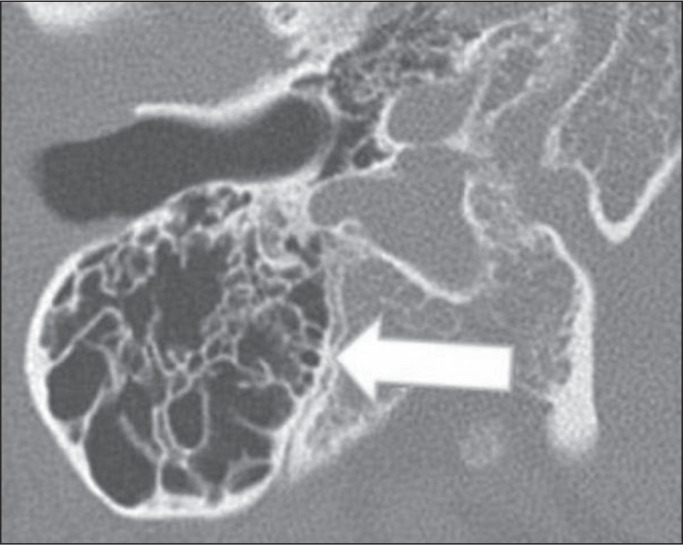
Occipitomastoid suture in a 10-year-old boy. Unenhanced axial CT of the temporal bone, showing a normal occipitomastoid suture (arrow).

The occipitomastoid suture is located between the occipital bone and the mastoid process of the temporal bone (Figure 10). It shows continuity with the lambdoid suture. The mastoid foramen can be located in the occipitomastoid suture. It is not uncommon to mistake it for a skull base fracture on axial CT scans, particularly when it is in an asymmetrical position. The mean age at which occipitomastoid suture closure occurs is 16 years. To distinguish the occipitomastoid suture from a fracture, one should know its normal location and two-sided nature. The sharp, nonsclerotic edges and angulation presented by cranial fractures are important criteria distinguishing them from sutures^(23,24)^.

#### Petroclival fissure

**Figure 11:**
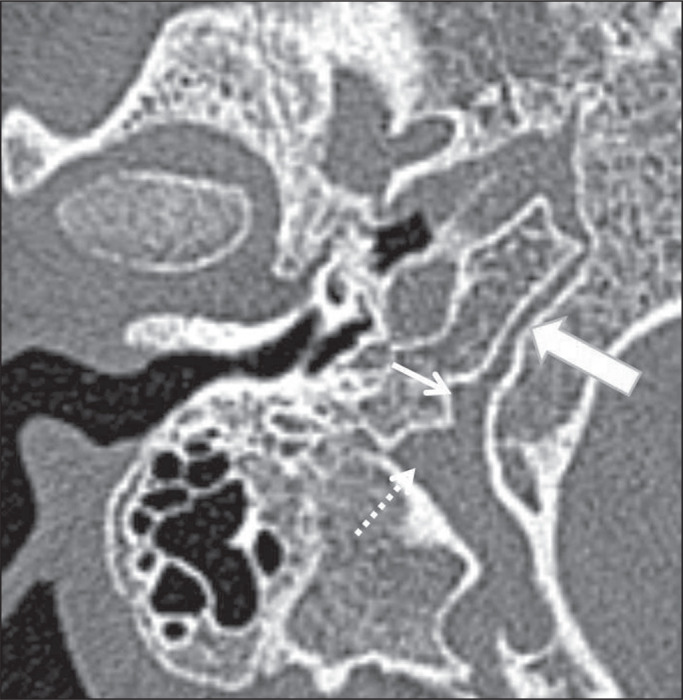
Petroclival fissure in a 24-year-old man. Unenhanced axial CT of the temporal bone, showing the petroclival fissure (thick arrow) in close proximity to the pars nervosa (thin arrow) of the jugular foramen (dotted arrow).

The clivus and the petrous portions of each temporal bone form the petroclival fissure, located in the anterior part of the posterior cranial fossa. It originates from the petrous apex, enlarges in the posterior-inferior direction, and extends to the neural structures of the jugular foramen (Figure 11). It runs from the cavernous sinus to the inferior petrosal sinus. The petroclival region is complex and difficult to access surgically. Formed by the union of the sphenoid, temporal, and occipital bone, it is a site from which intradural and extradural tumors extend. Lesions originating from the petroclival region can extend to the foramen magnum, jugular foramen, cerebellopontine angle, petrous apex, tentorial opening, temporal fossa, cavernous sinus, or Meckel’s cave^(25,26)^.

#### Sphenosquamosal suture

**Figure 12:**
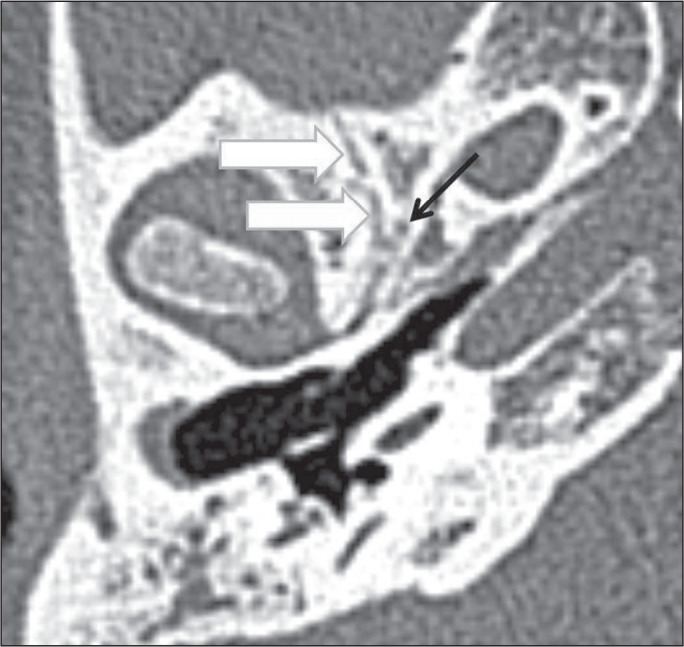
Sphenosquamosal suture in a 10-year-old boy. Unenhanced axial CT of the temporal bone, showing a normal sphenosquamosal suture (white arrows). It is typically located lateral to the foramen spinosum (black arrow).

The sphenosquamosal suture extends vertically and bilaterally between the sphenoid and temporal bones. It is located between the posterior edge of the greater wing of the sphenoid bone and the anterior margin of the squamous part of the temporal bone, lateral to the foramen spinosum (Figure 12). It typically undergoes fusion at 6-10 years of age^(23,27)^.

#### Petromastoid canal

**Figure 13:**
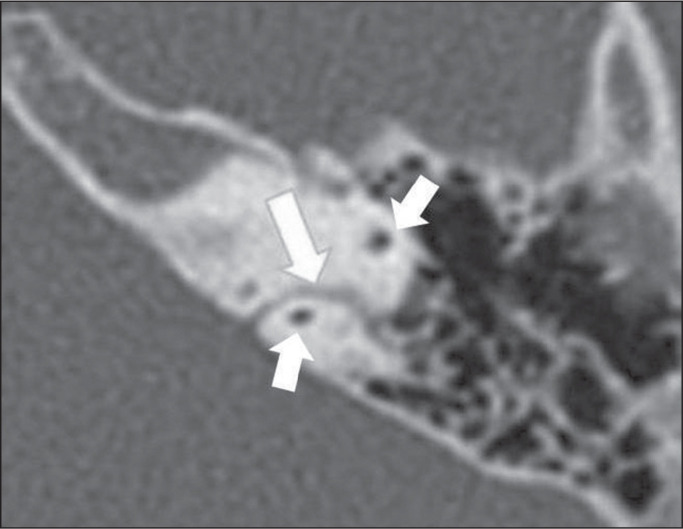
Petromastoid canal. Unenhanced axial CT of the temporal bone, showing a type II petromastoid canal (major arrow) passing between the two arches of the superior semicircular canal (minor arrows).

The petromastoid canal has a convex anterior course between the two sides of the superior semicircular canal (Figure 13). It unites the posterior fossa and the mastoid antrum. It contains the subarcuate artery and the subarcuate vein. It plays a role in the intracranial spread of mastoid infections. On CT, it can be easily mistaken for a fracture line^(7,18)^. Four types of petromastoid canal have been defined^(28)^: type I (undetectable); type II (< 0.5 mm in width); type III (0.5-1.0 mm in width); and type IV (> 1 mm in width).

#### Hiatus of the facial canal

**Figure 14:**
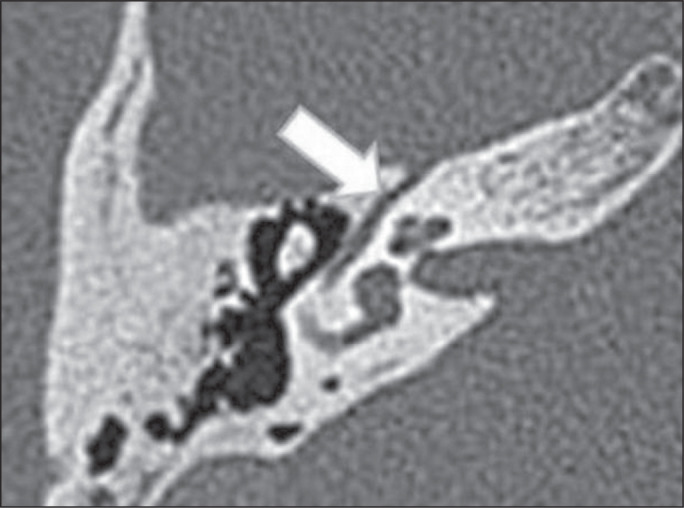
Hiatus of the facial canal in a 42-year-old man. Unenhanced axial CT of the temporal bone, showing the hiatus on the ventral surface of the petrous bone (arrow). It is continuous with the geniculate ganglion.

The hiatus of the facial canal is an anatomical cavity that allows the passage of the greater superficial petrosal nerve and the petrosal branch of the middle meningeal artery into the middle cranial fossa. It is in continuation with the geniculate ganglion and is located on the anterior surface of the petrous portion of the temporal bone (Figure 14). It also contains the greater superficial petrosal nerve^(7,18)^.

#### Singular canal

The singular canal, also known as the foramen singulare or singular foramen, extends from the posterior wall of the internal acoustic canal to the junction of the posterior semicircular canal and the vestibule. It contains the singular (posterior ampullary) nerve. The singular canal has a length of approximately 4 mm and terminates at the ampulla of the posterior semicircular canal. It is a major anatomical landmark in retrolabyrinthine surgery, and its injury puts the cochlear circulation at risk. It is a normal anatomical structure that can be mistaken for a temporal bone fracture on CT scans^(23,29)^.

#### Vestibular aqueduct

The vestibular aqueduct extends from the vestibule to the posterior surface of the petrous bone, running parallel to the latter. It contains the endolymphatic duct and sac. It normally has a diameter comparable to that of the posterior semicircular canal: < 1.5 mm at its midpoint. A smaller-than-normal vestibular aqueduct is a variation, whereas a larger-than-normal one is usually pathologic. Although a larger-than-normal vestibular aqueduct can be an isolated finding, it is often associated with incomplete partition type II, branchio-oto-renal syndrome, Pendred syndrome, or CHARGE syndrome. According to the Cincinnati criteria, a larger-than-normal vestibular aqueduct is defined as one with a diameter > 0.9 mm at its midpoint and > 1.9 mm at its opercular segment^(23,30)^.

#### Cochlear aqueduct

**Figure 15:**
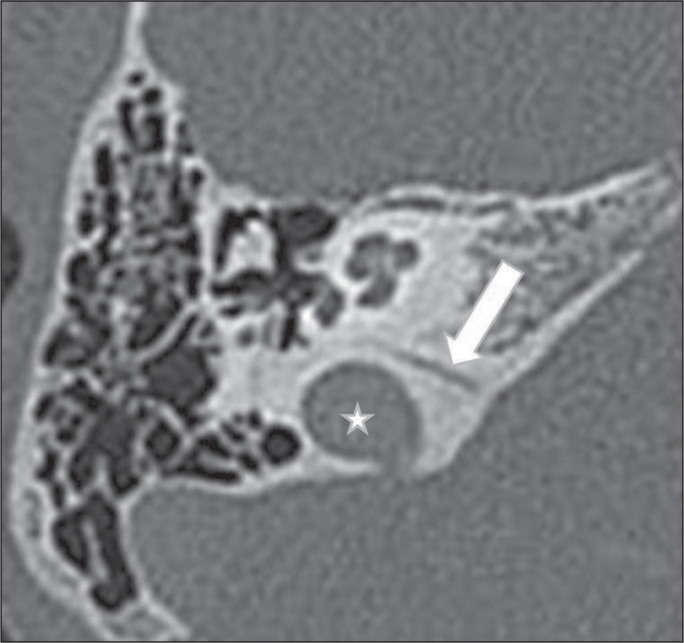
Cochlear aqueduct in a 45-year-old woman. Unenhanced axial CT of the temporal bone, showing the cochlear aqueduct coursing toward the cochlea (arrow). It can easily be confused with a fracture. (star; jugular foramen).

The cochlear aqueduct extends from the subarachnoid space to the basal turn of the cochlea, near the oval window (Figure 15). It contains the perilymphatic duct and serves as an entry point into the inner ear for infected cerebrospinal fluid. That can cause labyrinthitis ossificans in children with meningitis. The diameter of a normal cochlear aqueduct is 0-11 mm (mean, 4.5 mm); narrowing, particularly in its medial diameter, can be seen in Ménière’s disease^(23,31)^.

#### Inferior tympanic canaliculus and mastoid canaliculus

The inferior tympanic canaliculus is a small canal through which the tympanic branch of the glossopharyngeal nerve (Jacobson’s nerve) and the inferior tympanic artery course. The lesser inferior tympanic canaliculus is found on the bony ridge separating the carotid canal from the jugular fossa. The inferior tympanic canaliculus is located near the fossula petrosa, which contains the inferior ganglion of the glossopharyngeal nerve/petrous ganglion, from which the tympanic nerve originates. In cases of an abnormal internal carotid artery in which the petrous internal carotid artery is absent, the inferior tympanic artery is enlarged to form the proximal cranial internal carotid artery^(23,32)^.

The mastoid canaliculus originates from the vascular portion of the jugular foramen. It extends to the mastoid segment of the facial canal and contains Arnold’s nerve (the auricular branch of the vagus nerve).

It is important to have thorough knowledge of the course of Jacobson’s and Arnold’s nerves. They can be they mistaken for fractures, and paragangliomas tend to arise along their course^(23,33)^.

### Foramen of Huschke

**Figure 16:**
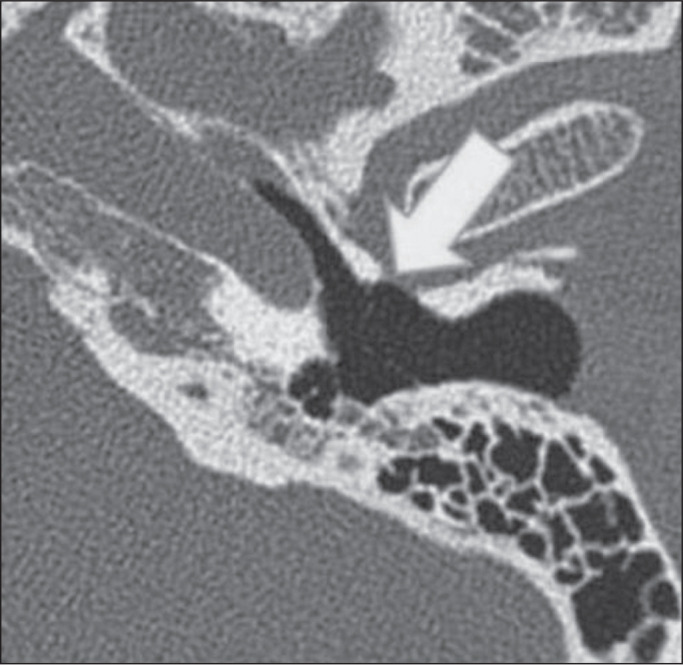
Foramen of Huschke in a 32-year-old woman. Unenhanced axial CT of the temporal bone, showing a foramen of Huschke with dehiscence between the temporomandibular joint and external auditory canal (arrow).

A foramen of Huschke is an asymptomatic incidental variant. It is a small ossification defect between the temporomandibular joint and the anteroinferior bony wall of the external auditory canal (Figure 16). It is usually closed by the age of 5 years. It varies in size and has an incidence of 5-20%. It can be unilateral or bilateral and is usually clinically unimportant. The incidence of a foramen of Huschke is 5-20%. There have been just a few reports of herniation from the temporomandibular fossa or fistulization from the salivary glands. It is important to have sufficient knowledge of this structure before performing skull base surgery, particularly temporomandibular joint arthroscopy. It can mimic a petrous temporal bone fracture, as well as potentially facilitating the spread of skull base infections and tumors^(34,35)^.

### Arachnoid granulations

Arachnoid granulations, also known as Pacchionian granulations, penetrate the arachnoid membrane and extend into the dura immediately beneath the vascular endothelium of the greater dural sinuses. In rare cases, they can be seen posterior to the temporal bone. On CT, they appear as filling defects with brain or spinal cord density, and they should be mentioned in radiology reports. They can cause cerebrospinal fluid leaks, otorrhea, and intracranial complications. Their main differential diagnosis is endolymphatic sac tumors. The absence of calcification and bone spicules is diagnostic for arachnoid granulations^(36,37)^.

### Asymmetric fatty marrow in the petrous apex

Asymmetric pneumatization of the petrous apex is a common, normal anatomic variant that results in asymmetric fatty marrow within the structure. It is a common finding on an MRI examination of the brain and skull base. Cases are often asymptomatic. The inner structure of the petrous apex is preserved. Asymmetric fatty marrow in the petrous apex has low attenuation on CT due to its low fat content. It appears as fat intensity in all MRI sequences. Its differential diagnosis should specifically include a cholesterol granuloma. The absence of dilation in the petrous apex and the preservation of its inner structure favor a finding of asymmetrical fatty marrow in the petrous apex^(17,38)^.

### Fluid-filled petrous air cells

Presence of trapped fluid in other mastoid cells usually accompanies fluid uptake in the petrous apex. The absence of dilation in the petrous apex, the absence of cortical disruption or trabecular erosion, and a preserved inner structure are diagnostic for fluid-filled petrous air cells. Petrous apex effusion can be mistaken for a cholesterol granuloma. However, some authors recommend follow-up within three years to determine its stability and to exclude an undetected cholesterol granuloma. Although contrast enhancement is not an expected sign in petrous apex effusions, a thin circumferential area of mucosal contrast enhancement can be observed in rare cases^(16,17)^.

### Superior semicircular canal dehiscence

Superior semicircular canal dehiscence is characterized by the weakening or complete absence of the bone structure overlying the superior semicircular canal (the arcuate eminence). In 10% of asymptomatic individuals, such dehiscence can be an incidental CT finding. Although dehiscence is most commonly monitored in the superior semicircular canal, it can also be seen in the lateral and posterior semicircular canals. It is often unilateral. In symptomatic patients, it is called superior semicircular canal dehiscence syndrome. The use of multiplanar reconstruction of thin-slice images acquired in multiple planes (including the Stenvers and Pöschl planes) increases diagnostic accuracy^(39)^.

## CONCLUSION

Although the temporal bone is only a small part of the human body, it is an important source of disease. The complex, variable anatomy of the temporal bone can pose significant diagnostic challenges for radiologists. Advances in imaging technology enable us to gain a better understanding of the anatomy of the temporal bone, as well as of the structural variations that potentially mimic diseases and of the true diseases that affect this region of the body.
